# Modulation of Murine Macrophage TLR7/8-Mediated Cytokine Expression by Mesenchymal Stem Cell-Conditioned Medium

**DOI:** 10.1155/2013/264260

**Published:** 2013-09-28

**Authors:** Takahiro Asami, Makoto Ishii, Hideki Fujii, Ho Namkoong, Sadatomo Tasaka, Kenichi Matsushita, Ken Ishii, Kazuma Yagi, Hiroshi Fujiwara, Yohei Funatsu, Naoki Hasegawa, Tomoko Betsuyaku

**Affiliations:** ^1^Division of Pulmonary Medicine, Department of Medicine, Keio University School of Medicine, 35 Shinanomachi, Shinjuku-ku, Tokyo 160-8582, Japan; ^2^Department of Microbiology and Immunology, Keio University School of Medicine, 35 Shinanomachi, Shinjuku-ku, Tokyo 160-8582, Japan; ^3^Second Department of Internal Medicine, Kyorin University School of Medicine, 6-20-2 Shinkawa, Mitaka-shi, Tokyo 181-8611, Japan; ^4^Department of Orthopedic Surgery, Keio University School of Medicine, 35 Shinanomachi, Shinjuku-ku, Tokyo 160-8582, Japan; ^5^Center for Infection Disease and Infection Control, Keio University School of Medicine, 35 Shinanomachi, Shinjuku-ku, Tokyo 160-8582, Japan

## Abstract

Increasing evidence suggests that mesenchymal stem cells (MSCs) play anti-inflammatory roles during innate immune responses. However, little is known about the effect of MSCs or their secretions on the ligand response of Toll-like receptor (TLR) 7 and TLR8, receptors that recognize viral single-stranded RNA (ssRNA). Macrophages play a critical role in the innate immune response to ssRNA virus infection; therefore, we investigated the effect of MSC-conditioned medium on cytokine expression in macrophages following stimulation with TLR7/8 ligands. After stimulation with TLR7/8 ligand, bone marrow-derived macrophages cultured with MSCs or in MSC-conditioned medium expressed lower levels of tumor necrosis factor (TNF) **α** and interleukin (IL) 6 and higher levels of IL-10 compared to macrophages cultured without MSCs or in control medium, respectively. The modulations of cytokine expression were associated with prostaglandin E_2_ (PGE_2_) secreted by the MSCs. PGE_2_ enhanced extracellular signal-related kinase (ERK) signaling and suppressed nuclear factor-**κ**B (NF-**κ**B) signaling. Enhanced ERK signaling contributed to enhanced IL-10 production, and suppression of NF-**κ**B signaling contributed to the low production of TNF-**α**. Collectively, these results indicate that MSCs and MSC-conditioned medium modulate the cytokine expression profile in macrophages following TLR7/8-mediated stimulation, which suggests that MSCs play an immunomodulatory role during ssRNA virus infection.

## 1. Introduction

Several lines of evidence confirm that the adaptive immune response plays a critical role in the effective control and clearance of various kinds of viruses [1, 2]. In addition, recent studies have demonstrated that innate immune responses are also important for viral clearance [3, 4]. Viral infection triggers various innate immune receptors known as pattern recognition receptors (PRRs), including Toll-like receptor (TLR), Nod-like receptor (NLR), and RIG-I-like receptor (RLR) [5]. The TLR family of PRRs was the first to be identified and to date is the most extensively studied. Both TLR7 and TLR8 are located in endosomes; they recognize the genomes of single-stranded RNA (ssRNA) viruses such as influenza virus and human immunodeficiency virus (HIV). Recognition by these receptors results in the activation of intracellular signaling by nuclear factor-*κ*B (NF-*κ*B) and mitogen activated protein kinases (MAPKs) through MyD88 activation, which in turn leads to the production of proinflammatory cytokines and chemokines and initiates various antiviral responses [5, 6]. 

In the lungs, macrophages as well as dendritic cells (DCs) constitute the first line of innate host defenses against viral infection by contributing to the inhibition of viral replication [4, 7]. For example, in influenza virus infection, the highly pathogenic H5N1 avian influenza virus and the H1N1 virus identified as the cause of the 2009 pandemic tend to directly infect alveolar macrophages in addition to epithelial cells [8]. Pharmacological depletion of macrophages reportedly reduces the rate of survival in animal models of influenza virus pneumonia, suggesting that macrophages are essential for effective immune responses to influenza virus infection [9]. During viral infection and associated TLR7/8 stimulation, macrophages produce various proinflammatory cytokines such as TNF-*α* and IL-6, which leads to an enhanced inflammatory response [10–12]. The production of another major immunomodulatory cytokine, IL-10, is also upregulated in TLR7/8-stimulated macrophages [12] and ssRNA virus-infected macrophages [13].

Mesenchymal stem cells (MSCs) are multipotent mesenchymal stromal cells that can be isolated from various tissues. They are capable of differentiating into mesodermal lineage cells such as bone, cartilage, and fat cells [14]. A growing body of evidence indicates that MSCs display unique immunomodulatory properties during inflammation in innate immune systems and that these cells are thus promising candidates for use in cell-mediated therapies for inflammatory diseases [15]. For example, MSC administration protects against experimental sepsis in mice as a result of enhanced production of the anti-inflammatory cytokine IL-10 by macrophages in response to MSC-secreted prostaglandin E_2_ (PGE_2_) [16]. Animal model studies have shown that the protective actions of MSCs also occur in response to acute lung injury induced by the TLR4 ligand lipopolysaccharide (LPS) [17], chronic obstructive pulmonary disease [18], pulmonary fibrosis [19], and bacterial pneumonia [20]. In addition, treatment with MSCs has been shown to improve lung function in a human *ex vivo*-perfused TLR4-mediated acute lung injury (ALI) model [21]. Although little is known about the role that MSCs may play in the immune response to viruses such as influenza viruses, the mechanism of MSC-induced immunomodulation has been studied. Both cell-cell contact and soluble factors secreted from MSCs, such as PGE_2_ [16], indoleamine 2,3 dioxygenase (IDO) [22], transforming growth factor beta-1 (TGF-*β*1) [23], hepatocyte growth factor (HGF) [24], inducible nitric-oxide synthase (iNOS) [25], and heme oxygenase-1 (HO1) [26], contribute to the modulation of immune responses [14].

In the present study, we hypothesized that MSCs and their soluble factors may modulate the cytokine expression induced by the activation of TLR7/8-mediated signaling, a major signaling pathway in innate immunity during ssRNA virus infections. To investigate this hypothesis, we focused on macrophages, which are critical components of the innate immune response to viral infection. Here, we investigated the effect of MSCs and MSC-conditioned medium on bone marrow-derived macrophages (BMDMs) during TLR7/8 stimulation. We found that TLR7/8-mediated expression of TNF-*α*, IL-6, and IL-10 is modulated by enhancement of extracellular signal-related kinase (ERK)-mediated signaling and suppression of NF-*κ*B-mediated signaling in response to PGE_2_ secreted by MSCs.

## 2. Materials and Methods

### 2.1. Reagents

R848 (TLR7/8 ligand) and loxoribine (TLR7 ligand) were purchased from InvivoGen (San Diego, CA, USA). The prostaglandin receptor EP2 antagonist AH6809 and EP4 antagonist GW 627368X were purchased from Cayman (Ann Arbor, MI, USA). The MEK/ERK inhibitor U0126, antibodies against the phosphorylated form of ERK, p38, c-Jun N-terminal kinase (JNK), and total inhibitor of kappa B (I*κ*B)-*α*, as well as horseradish peroxidase-conjugated rabbit or mouse IgG secondary antibodies were purchased from Cell Signaling Technology (Danvers, MA, USA). Anti-*β*-actin antibody was purchased from Sigma-Aldrich (St. Louis, MO, USA).

### 2.2. Culture of Mesenchymal Stem Cells

Human MSCs were obtained from Lonza (Allendale, NJ, USA), cultured in mesenchymal stem cell basal medium (MSCBM) supplemented with MSCGM SingleQuots (Lonza), and subcultured every 3-4 days by using fresh media. The culture medium was collected and centrifuged at 600 ×g for 10 min. The supernatant was used in further experiments as MSC-conditioned medium, while cell-free medium incubated under the same conditions as the MSC-conditioned medium served as a control. All media were kept at −80°C until use.

### 2.3. Culture of Murine Bone Marrow-Derived Macrophages

Bone marrow cells were harvested from 8- to 10-week-old male C57/B6 mice (Charles River Laboratories Japan, Inc., Yokohama, Japan) by flushing the femur and tibia with RPMI 1640 medium. Recovered cells were then cultured in bone marrow cell medium (20% FCS, 30% L-cell supernatant, 2 mM l-glutamine, 1% penicillin/streptomycin, and 0.25 *μ*g/mL amphotericin B in RPMI 1640). Fresh bone marrow cell medium was added on day 3. On day 6, the adherent cells were replated in RPMI 1640 medium supplemented with 10% FCS, 2 mM l-glutamine, and 1% penicillin/streptomycin for use as BMDMs. On day 7, the medium was removed and replaced with either MSC-conditioned medium, or control medium and the cells were incubated for 1 h, after which they were stimulated with either R848 or loxoribine.

### 2.4. Enzyme-Linked Immunosorbent Assay

Levels of TNF-*α*, IL-6, GM-CSF, IL-12 p70, and IL-10 were determined using a DuoSet ELISA Kit (R&D Systems, Minneapolis, MN, USA), and PGE_2_ levels were measured using an ELISA kit (Cayman), according to the manufacturer's instructions.

### 2.5. Quantitative Real-Time PCR

Total RNA was isolated using RNeasy Mini kits (QIAGEN, Valencia, CA, USA) and reverse transcribed using High Capacity cDNA Reverse Transcription kits (Applied Biosystems, Foster City, CA, USA), according to the manufacturers' instructions. Quantitative real-time PCR (qRT-PCR) analysis with SYBR Green was performed on a 7500 Fast Real-Time PCR System (Applied Biosystems). The sequences of the primers were as follows: 5′-GGTCAAAGGTTTGGAAGCAG-3′ (forward) and 5′-TGTGAAATGCCACCTTTTGA-3′ (reverse) for *Il1b*; 5′-TCTCCGTTACTTGGGGACAC-3′ (forward) and 5′-CCACACTCAAGAATGGTCGC-3′ (reverse) for *Cxcl1*; 5′-GTGGAATCTTCCGGCTGTAG-3′ (forward) and 5′-ACCATGACACTCTGCAACCA-3′ (reverse) for *Ccl3*; 5′-CCACTTCTTCTCTGGGTTGG-3′ (forward) and 5′-GTGCCCACGTCAAGGAGTAT-3′ (reverse) for *Ccl5*; and 5′-TTGATGGCAACAATCTCCAC-3′ (forward) and 5′-CGTCCCGTAGACAAAATGGT-3′ (reverse) for *Gapdh*, which was used as a loading control.

### 2.6. Immunoblotting

Following R848 stimulation, BMDMs were lysed in RIPA buffer (Thermo Fisher Scientific, Waltham, MA, USA) supplemented with protease inhibitor cocktail (Sigma-Aldrich) and phosphatase inhibitor (Thermo Fisher Scientific) at various time points. Lysed BMDMs were kept on ice for 30 min and then centrifuged at 15,000 ×g for 15 min. The supernatant was collected and stored at −80°C until use. The total protein concentration of each sample was determined using the bicinchoninic acid protein assay (Thermo Fisher Scientific). Equal amounts (10–30 *μ*g) of cell lysate were separated by SDS-PAGE (Bio-Rad, Hercules, CA, USA), and the proteins were then transferred onto polyvinyl difluoride membranes (Invitrogen, Carlsbad, CA, USA). After overnight incubation with each primary antibody, the membrane was washed, stained with horseradish peroxidase-conjugated rabbit or mouse IgG secondary antibody, and visualized using enhanced chemiluminescence detection reagents (ECL; GE Healthcare, Piscataway, NJ, USA). The images were analyzed using ImageJ 1.37v (National Institutes of Health, Bethesda, MD, USA).

### 2.7. RelA Transfection Study

BMDMs were transfected with the RelA cFlag pcDNA3 plasmid (gift from Professor Stephen Smale) or pcDNA3.1 plasmid (gift from Professor Stephen Smale) using the FuGENE6 Transfection Reagent (Promega, Madison, WI, USA), according to the manufacturer's instructions. At 24 h after transfection, the cells were cultured for 1 h in either MSC-conditioned medium or control medium, after which they were incubated with either TLR7/8 ligand (R848) or vehicle, without changing the medium.

### 2.8. Statistical Analysis

Data are expressed as the mean ± SEM. Differences were analyzed for statistical significance using an unpaired *t*-test or ANOVA, followed by Tukey's test for multiple comparisons. *P* values less than 0.05 were considered statistically significant.

## 3. Results

### 3.1. MSCs Modulate Cytokine Levels after TLR7/8 Ligand Stimulation

**Figure 1:**
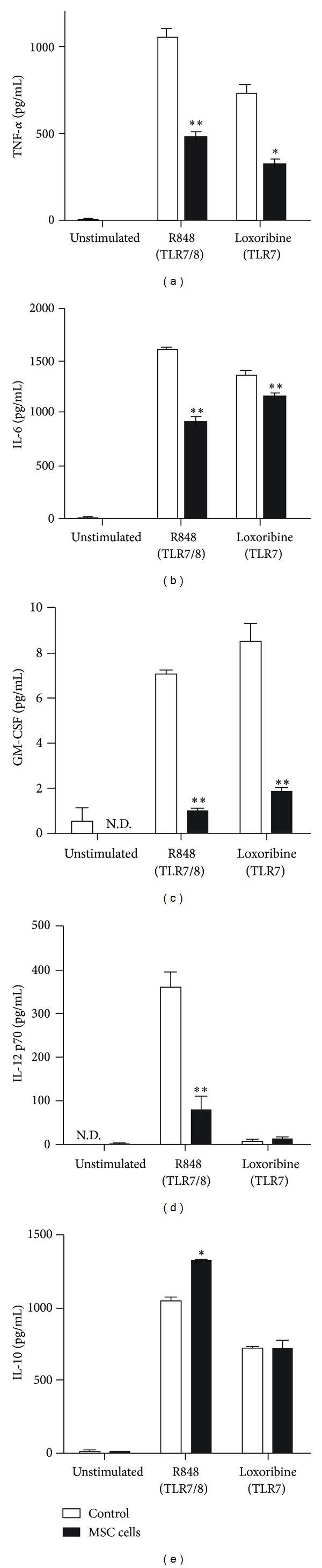
Modulation of cytokine expression by BMDMs cocultured with MSCs and then stimulated with TLR7/8 ligands. BMDMs (0.4 × 10^6^ cells/well) were incubated with or without MSCs (0.4 × 10^6^ cells/well) in 24-well plates. After 1 h, the cells were incubated with R848 (TLR7/8 ligand, 10 *μ*g/mL) or loxoribine (TLR7 ligand, 300 *μ*g/mL) for 24 h. The levels of murine TNF-*α*, IL-6, GM-CSF, IL-12 p70, and IL-10 in the culture supernatant were determined using ELISA. Data are expressed as the mean ± SEM. *n* = 3 in each group. *P < 0.05 and **P < 0.01, compared to the control group. N.D., not detected. Results are representative of 3 independent experiments.

To investigate the effect of MSCs on cytokine production by BMDMs following TLR7/8 ligand stimulation, we cocultured MSCs and BMDMs and examined cytokine production following stimulation with either the TLR7/8 (R848) or TLR7 (loxoribine) ligand. MSCs inhibited the production of TNF-*α*, IL-6, and GM-CSF by BMDMs after R848 and loxoribine stimulation. The level of IL-12 p70 was lower in cocultured BMDMs after R848 stimulation, but not after loxoribine stimulation. The level of IL-10 was higher in cocultured BMDMs after R848 stimulation, but not after loxoribine stimulation (Figure 1). 

### 3.2. MSC-Conditioned Medium Modulates Cytokine Levels after TLR7/8 Ligand Stimulation

**Figure 2:**
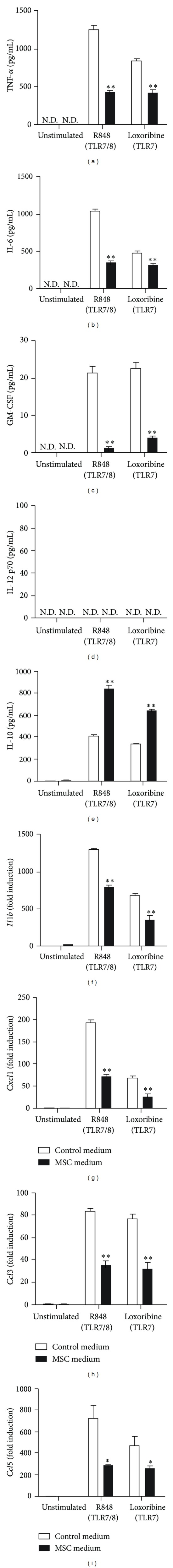
Modulation of cytokine expression by BMDMs incubated in MSC-conditioned medium and then stimulated with TLR7/8 ligands. BMDMs were preincubated for 1 h in MSC-conditioned medium or control medium and then incubated with R848 (TLR7/8 ligand, 10 *μ*g/mL) or loxoribine (TLR7 ligand, 300 *μ*g/mL) for 24 h. (a–e) The levels of murine TNF-*α* (a), IL-6 (b), GM-CSF (c), IL-12 p70 (d), and IL-10 (e) in the culture supernatant were determined using ELISA. Data are expressed as the mean ± SEM. *n* = 3 in each group. *P < 0.05 and **P < 0.01, compared to the control medium group. N.D., not detected. (f–i) The levels of *Il1b *(f), *Cxcl1 *(g), *Ccl3 *(h), and *Ccl5 *(i) mRNA were determined using qRT-PCR. Data are expressed as fold increase over the level in the control medium group without TLR7/8 ligand stimulation (mean ± SEM). *n* = 3 in each group. *P < 0.05 and **P < 0.01, compared to the control medium group. Results are representative of 3-4 independent experiments.

Next, we examined whether MSCs produce soluble factors that modulate cytokine production by macrophages stimulated with TLR7/8 ligands. BMDMs were preincubated in either MSC-conditioned or control medium and then stimulated with the TLR7/8 (R848) or TLR7 (loxoribine) ligand. The production of TNF-*α*, IL-6, and GM-CSF following R848 or loxoribine stimulation was significantly lower in BMDMs preincubated in MSC-conditioned medium than in BMDMs preincubated in control medium (Figures 2(a)–2(c)). IL-12 p70 was not detected in either case (Figure 2(d)). In contrast, R848- and loxoribine-stimulated BMDMs preincubated in MSC-conditioned medium produced higher levels of IL-10 than did R848- and loxoribine-stimulated BMDMs preincubated in control medium (Figure 2(e)). These results indicate that MSCs secrete factors that suppress the production of TNF-*α*, IL-6, and GM-CSF and enhance the production of IL-10 by TLR7/8 ligand-stimulated BMDMs.

We also examined the expression of other virus-related cytokines. The production of mRNA encoding *Il1b, CXC ligand (Cxcl)1, CC ligand (Ccl)3, *and* Ccl5* was suppressed in both R848- and loxoribine-stimulated BMDMs preincubated in MSC-conditioned medium (Figures 2(f)–2(i)). 

### 3.3. PGE_2_/PGE_2_ Receptors Contribute to the Modulation of Cytokine Production in MSC-Conditioned Medium

**Figure 3:**
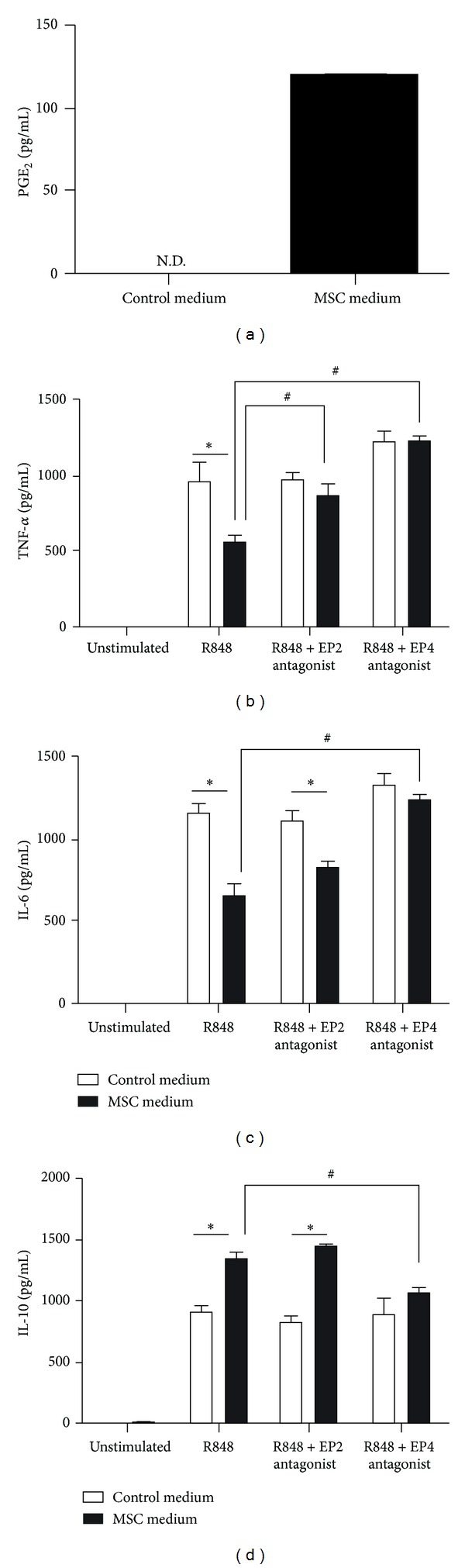
Contribution of PGE_2_/EP to the modulation of cytokine expression in BMDMs incubated in MSC-conditioned medium and then subjected to R848 stimulation. (a) PGE2 levels in MSC-conditioned medium or control medium were measured by ELISA. N.D., not detected. (b–d) BMDMs were incubated in MSC-conditioned medium or control medium with EP2 antagonist (AH6809, 10 *μ*M) or EP4 antagonist (GW 627368X, 10 *μ*M). DMSO was used as the vehicle for each EP antagonist. After 1 h, R848 (TLR7/8 ligand, 10 *μ*g/mL) was added, and the BMDMs were incubated for an additional 24 h. The levels of TNF-*α* (b), IL-6 (c), and IL-10 (d) were determined using ELISA. *P < 0.05, compared to the control medium group. ^#^ *P* < 0.05, compared to the R848-stimulated cells (without EP antagonist). Data are expressed as the mean ± SEM. *n* = 3 in each group. Results are representative of 3 independent experiments.

PGE_2_ is a major immunomodulator secreted by MSCs [14], and a previous study demonstrated that PGE_2_ secreted from MSCs contributes to enhanced IL-10 production by macrophages [16]. Therefore, we examined whether PGE_2_ contributes to the immunomodulatory effect of MSC-conditioned medium on TLR7/8 ligand (R848)-stimulated BMDMs. We first confirmed that PGE_2_ was present in MSC-conditioned medium, but not in control medium (Figure 3(a)). We then measured the level of PGE_2_ in the supernatant of BMDMs before and after TLR7/8 ligand (R848) stimulation. The level was below the detection limit, suggesting that PGE_2_ is not secreted by macrophages after TLR7/8 ligand stimulation (data not shown).

Next, BMDMs were preincubated for 1 h with the prostaglandin E2 receptor EP2 or EP4 antagonist in MSC-conditioned or control medium, after which the cells were stimulated with R848. The results are shown in Figures 3(b)–3(d). The level of TNF-*α* produced by cells preincubated with the EP2 or EP4 receptor antagonist was significantly higher than that produced by cells stimulated with R848 alone (Figure 3(b)). The level of IL-6 increased significantly in BMDMs preincubated with the EP4 antagonist in MSC-conditioned medium, but not in cells preincubated with the EP2 antagonist (Figure 3(c)). In addition, the observed enhancement in IL-10 production was partly abrogated by the EP4 antagonist, but not by the EP2 antagonist (Figure 3(d)). These results indicate that the observed decreases in the expression of TNF-*α* and IL-6 and the observed increase in the expression of IL-10 were due, at least in part, to PGE_2_ secreted by MSCs.

### 3.4. MSC-Conditioned Medium Enhances ERK Signaling and Suppresses NF-*κ*B Signaling in TLR7/8 Ligand-Stimulated BMDMs

**Figure 4:**
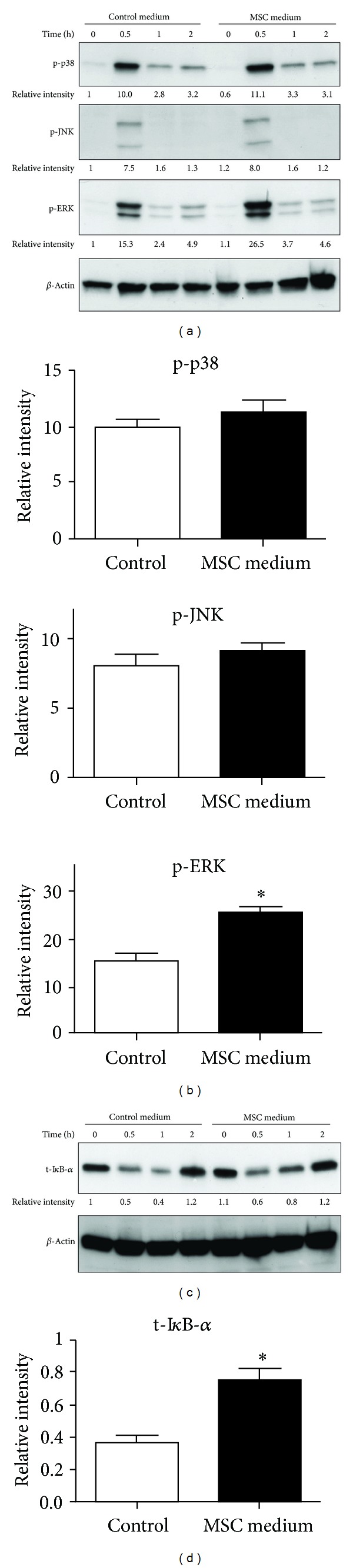
Enhanced ERK and suppressed NF-*κ*B signaling in BMDMs incubated in MSC-conditioned medium and then subjected to R848 stimulation. BMDMs were preincubated for 1 h in MSC-conditioned medium or control medium, after which R848 (TLR7/8 ligand, 10 *μ*g/mL) was added. Cells were further incubated for the indicated times. (a) The levels of p-p38, p-JNK, and p-ERK were determined by immunoblotting. Beta-actin was used as a loading control. Relative band intensities ware quantified by densitometry analysis. (b) The data of (a) at 0.5* *h after R848 stimulation obtained by densitometric analysis are shown as the mean ± SEM of 3 independent experiments. (c) The level of total I*κ*B-*α* was determined by immunoblotting. Beta-actin was used as a loading control. Relative band intensities ware quantified by densitometry analysis. (d) The data of (c) at 1 h after R848 stimulation obtained by densitometric analysis are shown as the mean ± SEM of 3 independent experiments. *P < 0.05, compared to the control medium group.

Because MSC-conditioned medium modulated cytokine expression, especially after R848 stimulation, we examined the effect of MSC-conditioned medium on signaling mediated by the MAPK and NF-*κ*B pathways, both of which play important roles in TLR-mediated cytokine expression [27]. The level of phosphorylated (p)-ERK was elevated 0.5 h after R848 stimulation in BMDMs preincubated in MSC-conditioned medium compared to the level in cells preincubated in control medium (Figures 4(a) and 4(b)). At 1 h after R848 stimulation, the level of total I*κ*B-*α*, the inhibitory protein of NF-*κ*B, was higher in BMDMs preincubated in MSC-conditioned medium than in cells preincubated in control medium (Figures 4(c) and 4(d)). These results indicate that MSC-conditioned medium enhances signaling mediated by ERK and suppresses signaling mediated by NF-*κ*B in BMDMs stimulated with R848. 

### 3.5. Involvement of ERK Signaling in Modulation of IL-10 Expression in BMDMs Preincubated in MSC-Conditioned Medium

**Figure 5:**
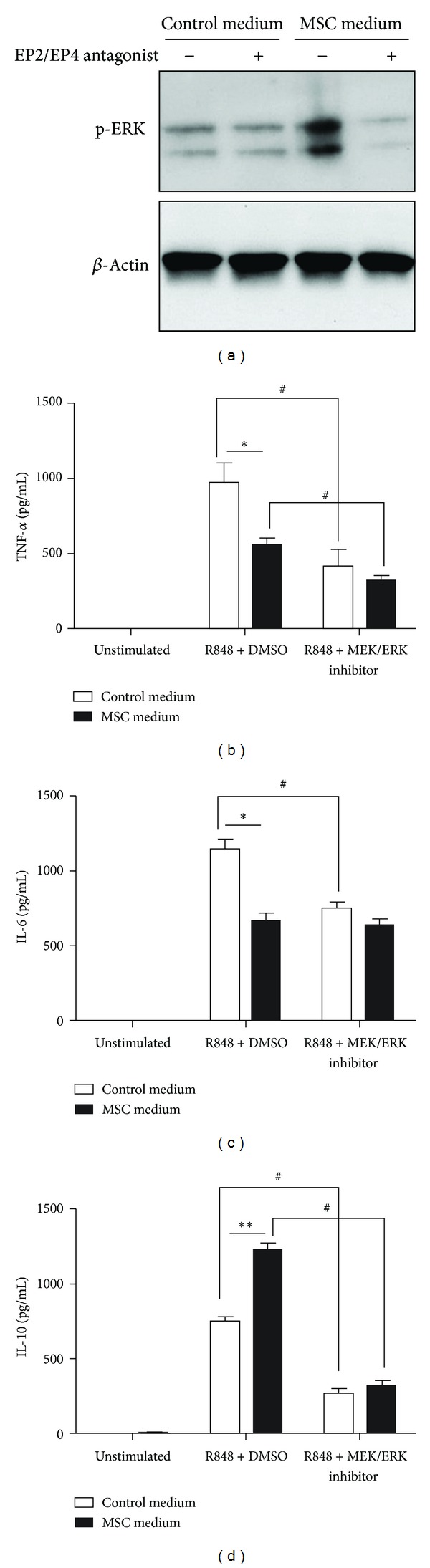
ERK signaling is partially responsible for the modulated expression of IL-10, but not that of TNF-*α* or IL-6, in BMDMs preincubated in MSC-conditioned medium followed by R848 stimulation. (a) BMDMs were preincubated in MSC-conditioned medium or control medium with or without a cocktail consisting of an EP2 antagonist (AH6809, 10 *μ*M) and an EP4 antagonist (GW 627368X, 10 *μ*M). DMSO was used as a vehicle. After 1 h, R848 (TLR7/8 ligand, 10 *μ*g/mL) was added, and BMDMs were incubated for an additional 0.5 h. The p-ERK level was determined by immunoblotting. Beta-actin was used as a loading control. Results are representative of 3 independent experiments. (b–d) BMDMs were preincubated in MSC-conditioned medium or control medium with or without MEK/ERK inhibitor (U-0126, 10 *μ*M). DMSO was used as the vehicle. After 1 h, R848 (TLR7/8 ligand, 10 *μ*g/mL) was added, and BMDMs were incubated for an additional 24 h. The levels of TNF-*α* (a), IL-6 (c), and IL-10 (d) were determined using ELISA. *P < 0.05 and **P < 0.01, compared to the control medium group. ^#^ *P* < 0.05, compared to the R848-stimulated cells (without MEK/ERK inhibitor). Data are expressed as the mean ± SEM. *n* = 3 in each group. Results are representative of 3 independent experiments.

To determine whether the PGE_2_ present in MSC-conditioned medium is responsible for the observed enhancement of ERK signaling in R848-stimulated BMDMs, we measured the level of p-ERK after R848 stimulation in BMDMs cultured in MSC-conditioned medium or control medium with or without a cocktail composed of the EP2 and EP4 antagonists. Treatment with the EP2/EP4 antagonist cocktail abrogated the increase in the level of p-ERK in R848-stimulated BMDMs cultured in MSC-conditioned medium (Figure 5(a)), suggesting that PGE_2_/EP plays a role in increasing the level of p-ERK in BMDMs incubated in MSC-conditioned medium.

We then examined whether enhanced p-ERK expression contributes to the observed suppression of TNF-*α* and IL-6 expression and enhancement of IL-10 expression in BMDMs incubated in MSC-conditioned medium. For this experiment, BMDMs were preincubated in MSC-conditioned or control medium containing the MEK/ERK inhibitor U0126 or dimethyl sulfoxide (DMSO, as a vehicle control), after which the cells were stimulated with R848. In cells incubated in the control medium, addition of U0126 resulted in a decrease in the levels of TNF-*α*, IL-6, and IL-10 after R848 stimulation (Figures 5(b)–5(d)), suggesting that MEK/ERK activation is essential for the production of these cytokines in R848-stimulated BMDMs. As shown previously in Figure 2(a), we confirmed that incubation in MSC-conditioned medium results in a significant decrease in the levels of TNF-*α* and IL-6 and a significant increase in the level of IL-10 after R848 stimulation (Figures 5(b)–5(d)). In cells incubated in MSC-conditioned medium, addition of U0126 did not increase the production of TNF-*α* and IL-6 after R848 stimulation (Figures 5(b) and 5(c)), suggesting that the stimulation of p-ERK expression induced by MSC-conditioned medium does not contribute to the decreased expression of TNF-*α* or IL-6. On the other hand, treatment with U0126 resulted in a significant decrease in IL-10 production in R848-stimulated BMDMs incubated in MSC-conditioned medium. The IL-10 level in these cells was comparable to that in R848-stimulated/U0126-treated BMDMs cultured in control medium (Figure 5(d)). These results suggest that enhanced p-ERK expression contributes at least partially to the enhancement of IL-10 production in BMDMs cultured in MSC-conditioned medium.

### 3.6. Involvement of NF-*κ*B Signaling in Modulation of TNF-*α* Expression in BMDMs Incubated in MSC-Conditioned Medium

**Figure 6:**
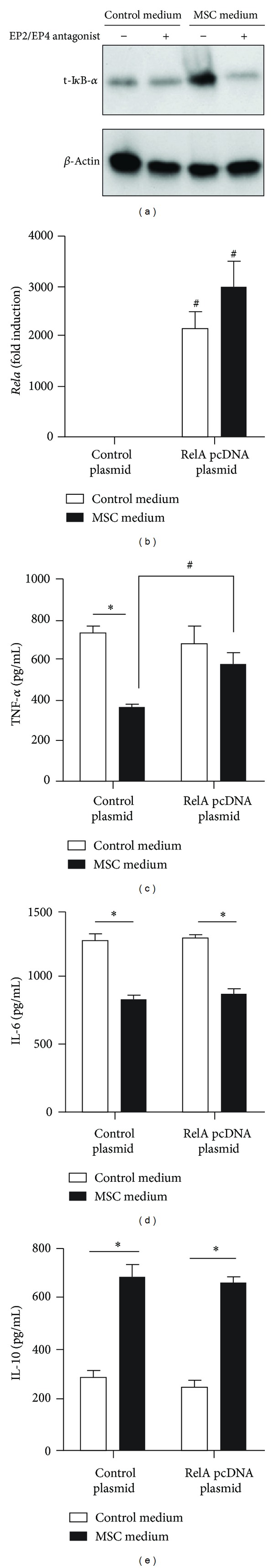
NF-*κ*B signaling is partially responsible for the modulated expression of TNF-*α*, but not that of IL-6 or IL-10, in BMDMs preincubated in MSC-conditioned medium and then subjected to R848 stimulation. (a) BMDMs were incubated in MSC-conditioned medium or control medium with or without a cocktail consisting of an EP2 antagonist (AH6809, 10 *μ*M) and EP4 antagonist (GW 627368X, 10 *μ*M). DMSO was used as the vehicle. After 1 h, R848 (TLR7/8 ligand, 10 *μ*g/mL) was added, and BMDMs were incubated for an additional 1 h. The level of total I*κ*B-*α* was determined by immunoblotting. Beta-actin was used as a loading control. Results are representative of 3 independent experiments. (b–e) BMDMs were transfected with RelA cFlag pcDNA3 plasmid or pcDNA3.1 plasmid (as a control) using FuGENE6 Transfection Reagent. After 24 h, the cells were incubated for 1 h in either MSC-conditioned medium or control medium, after which they were stimulated with TLR7/8 ligand (R848) or vehicle for 24 h in either MSC-conditioned medium or control medium. The level of *Rela* mRNA was determined using qRT-PCR (b). Data are expressed as the fold increase over the level of *Rela* in control plasmid-transfected cells cultured in control medium without R848 stimulation (mean ± SEM). ^#^ *P* < 0.05, compared to the control plasmid-transfected cells. The levels of TNF-*α* (c), IL-6 (d), and IL-10 (e) in the supernatant were determined using ELISA. *P < 0.05, compared to the control medium group. ^#^ *P* < 0.05, compared to the control plasmid-transfected cells. Data are expressed as the mean ± SEM. *n* = 3 in each group. Results are representative of 3 independent experiments.

To determine whether the suppression of NF-*κ*B signaling observed in R848-stimulated BMDMs incubated in MSC-conditioned medium is PGE_2_-dependent, we measured the total I*κ*B-*α* level in BMDMs cultured in MSC-conditioned or control medium with or without a cocktail composed of the EP2 and EP4 antagonists. The level of total I*κ*B-*α* was significantly lower in BMDMs cultured in MSC-conditioned medium containing the EP2/EP4 antagonist cocktail than in cells cultured in MSC-conditioned medium without the antagonist cocktail, suggesting that PGE_2_/EP plays a role in the inhibition of total I*κ*B-*α* degradation (i.e., suppression of NF-*κ*B signaling) in cells cultured in MSC-conditioned medium (Figure 6(a)).

We also investigated whether suppression of NF-*κ*B signaling contributes to the suppression of TNF-*α* and IL-6 expression and enhancement of IL-10 expression by transfecting BMDMs with either the RelA cFlag pcDNA3 plasmid or a control c-Flag pcDNA3 plasmid. Transfected cells were then incubated in either MSC-conditioned medium or control medium and stimulated with R848.As shown in Figure 6(b), *Rela* mRNA production was strongly induced in cells transfected with the RelA cFlag pcDNA3 plasmid. In cells incubated in the control medium, RelA overexpression resulted in no significant increase in the levels of TNF-*α*, IL-6, or IL-10 after R848 stimulation (Figures 6(c)–6(e)). In contrast, RelA overexpression enhanced the expression of TNF-*α* (but not IL-6 or IL-10) in cells incubated in MSC-conditioned medium (Figures 6(c)–6(e)). The production of TNF-*α* in RelA-transfected cells cultured in MSC-conditioned medium and control medium was comparable (Figure 6(c)). These results indicate that the expression of TNF-*α* in BMDMs incubated in MSC-conditioned medium is mediated at least in part by NF-*κ*B signaling.

## 4. Discussion

In this study, we investigated the effects of MSCs and MSC-conditioned medium on macrophages during TLR7/8-mediated immune responses and demonstrated the following salient findings: (1) TLR7/8-mediated cytokine expression, including that of TNF-*α*, IL-6, and IL-10, in macrophages is modulated by coincubation with MSCs; (2) soluble factors secreted by MSCs modulate TLR7/8-mediated cytokine expression in macrophages; (3) PGE_2_ secreted by MSCs is involved in this modulation; (4) ERK signaling is enhanced and NF-*κ*B signaling is suppressed after TLR7/8 ligand stimulation when macrophages are cultured in MSC-conditioned medium, and these effects are primarily due to PGE_2_; (5) ERK signaling is involved in enhanced IL-10 production in macrophages cultured in MSC-conditioned medium; and (6) NF-*κ*B signaling is involved in suppressed TNF-*α* (but not IL-6) expression in macrophages cultured in MSC-conditioned medium.

A growing body of evidence indicates that MSCs have immunomodulatory roles in innate immune responses; therefore, MSC-based therapies are increasingly viewed as promising means of treating various inflammatory diseases, such as sepsis [16], acute lung injury [17], and bacterial pneumonia [20]. In the present study, we hypothesized that MSCs may modulate TLR7/8-mediated signaling, a major signaling pathway activated by infection with ssRNA viruses such as influenza virus and HIV.

Macrophages are one of the most important cellular components of the inflammatory and innate immune responses that occur in the lung following infection with microorganisms such as bacteria and viruses [4, 28]. Recent reports indicate that macrophages play a pivotal role in the immunomodulation of MSCs during TLR4 (LPS)-mediated innate immune responses [17, 20]; however, the effect of MSCs on innate immune responses to ligands for TLR7/8, the receptors that recognize viral single-stranded RNA, is largely unknown. Therefore, we focused our investigation on the effect of MSCs on TLR7/8-mediated cytokine expression in macrophages.

Our results indicate that TLR7/8-mediated induction of proinflammatory cytokine expression (e.g., TNF-*α* and IL-6) was suppressed in BMDMs cultured in MSC-conditioned medium, suggesting that MSC-conditioned medium played an anti-inflammatory role in the present study. The significance of the induction of TNF-*α* and IL-6 expression following the TLR7/8-mediated response to ssRNA virus infection is not fully known. Infection with influenza virus, a major ssRNA virus, induces a significant increase in the production of TNF-*α* and IL-6 by macrophages, leading to enhanced inflammatory responses [11, 29]. In addition, increased TNF-*α* and IL-6 production can potentiate the severity of combined influenza A virus and bacterial infections [30, 31]. Another study demonstrated that the level of IL-6 expression correlates with disease severity in pediatric H1N1 infection [32]. Based upon these data, we speculate that the suppression of TLR7/8-mediated TNF-*α* and IL-6 expression by MSC-conditioned medium modulates the pathogenesis of ssRNA virus infection, including infection by influenza virus.

The expression of IL-10 was highly upregulated in BMDMs after TLR7/8 ligand stimulation in the present study, consistent with previous reports demonstrating that IL-10 expression is highly upregulated in macrophages following TLR7/8 ligand stimulation [33] and influenza virus infection [13]. Interestingly, we found that culturing BMDMs in MSC-conditioned medium increased the level of IL-10 after TLR7/8 ligand stimulation. The significance of TLR7/8-mediated induction of IL-10 expression in macrophages is controversial. However, given that IL-10 suppresses the induction of TNF-*α* and IL-6 expression in TLR ligand-stimulated macrophages [34], it is possible that IL-10 also inhibits the induction of TNF-*α* and IL-6 expression by macrophages, leading to the suppression of inflammatory responses observed in the present study.

In the presence of loxoribine (TLR7 ligand), IL-10 levels were comparable in BMDMs cultured alone and in BMDMs cocultured with MSCs. However, in the presence of loxoribine, the IL-10 level was higher in BMDMs cultured in MSC-conditioned medium than in cells cultured in control medium. A possible reason for the discrepancy is the effect of cell-cell contact between BMDMs and MSCs, which may modulate cell surface markers, change intracellular signaling, and modulate loxoribine-induced IL-10 production in the present study. 

The mechanism of MSC-mediated immunomodulation has been investigated. Soluble factors produced by MSCs as well as cell-cell contact are thought to be important for the immunomodulatory effects [14]. Various immunosuppressive factors secreted by MSCs mediate the immunomodulatory effects, including PGE_2_ [16], IDO [22], TGF-*β*1 [23], HGF [24], iNOS [25], and HO1 [26]. Among these factors, we focused on the role of PGE_2_ because PGE_2_, is a major secretory product of MSCs, and it can modulate cytokine expression, especially in macrophages [16].

Prostaglandin E_2_ is a major metabolite of arachidonic acid. It regulates inflammation and multiple functions of immune cells [35, 36]. Four PGE_2_ receptors have been identified: EP1, EP2, EP3, and EP4. The cAMP/PKA/CREB signaling pathway is activated through EP2 and EP4, both of which are responsible for the anti-inflammatory function of PGE_2_ [36]. The results of the present study indicate that the PGE_2_-EP2 and PGE_2_-EP4 pathways play important roles in the modulation of cytokine expression. The role of PGE_2_ in TNR7/8-mediated responses, including ssRNA virus infection, is not fully known; however, recent reports suggest that PGE_2_ protects against ssRNA virus infection *in vitro* [37, 38]. These results led us to speculate that PGE_2_ secreted by MSCs following ssRNA virus infection may play an immunoregulatory role *in vivo* by modulating cytokine expression. MSC-based therapy against ssRNA virus infection may be useful for patients with severe viral infection, such as those infected by the pandemic influenza 2009 H1N1 virus. In these cases, a cytokine storm can be lethal [39], and MSC-mediated suppression of proinflammatory cytokines (e.g., TNF-*α* and IL-6) protects against severe influenza virus infection. *In vivo* animal studies and clinical trial are required to fully understand the *in vivo* roles of MSCs.

Activation of TLR7/8 signaling through MyD88 results in the activation of MAPKs and the NF-*κ*B signaling pathway, leading to the expression of various cytokines [13]. Previous studies have demonstrated that PGE_2_ inhibits NF-*κ*B-mediated transcription [40, 41]. In addition, PGE_2_ reportedly enhances signaling via the ERK pathway [42]. Therefore, we examined the expression of signaling molecules associated with the MAPK and NF-*κ*B pathways. Interestingly, the expression of p-ERK, an activated form of ERK, was higher and the expression of total I*κ*B-*α*, an inhibitory protein of NF-*κ*B, was lower in BMDMs cultured in MSC-conditioned medium than in cells cultured in control medium, indicating that ERK signaling was activated and NF-*κ*B signaling was suppressed by the MSC-conditioned medium. In the present study, the EP2/EP4 antagonist cocktail decreased the level of p-ERK and total-I*κ*B-*α*, confirming that PGE_2_ contributes to enhanced ERK and suppressed NF-*κ*B signaling. We found that suppressed NF-*κ*B signaling was associated with suppressed TNF-*α* expression. We also found that enhanced ERK signaling was associated with enhanced IL-10 expression, consistent with a previous report indicating that ERK signaling is one of the primary pathways leading to IL-10 production in macrophages [43].

Macrophages can be phenotypically polarized into 2 main groups depending on the microenvironment: M1 (classical) or M2 (alternative) activated macrophages. TNF-*α* and IL-6, proinflammatory cytokines, are M1 macrophage markers, and IL-10 is an M2 macrophage marker [44]. Therefore, our results indicate that MSC or MSC-conditioned medium can polarize macrophage population toward an M2 phenotype. Other M1/M2 markers were not measured. A recent report indicates that MSCs and MSC-conditioned medium protect against LPS-induced acute lung injury by polarizing the macrophage population toward an M2 phenotype [45].

We used human-derived MSCs in the present study. These cells were used because results obtained using human MSCs may be more relevant to clinical settings and because little is known about the behavior of human MSCs during inflammatory responses, although the beneficial effects of human MSCs have been reported in studies of a mouse model of Gram-negative sepsis [46], LPS-induced ALI [47], and *Escherichia coli* pneumonia [48]. Suppression of TNF-*α* and IL-6 expression and enhancement of IL-10 expression were observed in the present study when R848-stimulated BMDMs were cultured in murine MSC-conditioned medium (data not shown), confirming that these immunomodulatory effects were not specific to human MSCs.

There are several limitations to the present study. First, the pathway responsible for the suppression of IL-6 expression in cells incubated in MSC-conditioned medium is unknown. Neither the ERK nor NF-*κ*B signaling pathways were responsible for the suppression of IL-6 expression. The Janus kinase/signal transducer and activator of transcription/suppressor of cytokine signaling and phosphoinositol-3-kinase pathways, both of which are important for IL-6 production [49, 50], could be responsible. Second, MSCs secrete soluble factors aside from PGE_2_ that can modulate TLR7/8-mediated signaling, such as TGF-*β* [23], which has been shown to suppress the production of proinflammatory cytokines in macrophages [51]. Further studies are required in order to obtain a complete understanding of the modulatory roles of MSCs.

In summary, we have demonstrated that MSC-conditioned medium has an immunomodulatory effect on macrophages. In macrophages incubated in MSC-conditioned medium, TLR7/8-mediated expression of the proinflammatory cytokines TNF-*α* and IL-6 was suppressed, and the expression of IL-10 was enhanced. These effects were mediated, in part, by PGE_2_. Suppression of NF-*κ*B signaling contributed to the suppression of TNF-*α* expression, and activation of ERK signaling contributed to enhanced IL-10 expression. These results indicate that MSC-conditioned medium modulates the cytokine expression profile of cells stimulated with TLR7/8. Our results also suggest that MSCs may play anti-inflammatory roles by modulating cytokine expression during infection by ssRNA viruses, such as influenza virus. Thus, MSCs are a potential therapeutic target for treating ssRNA virus infections.
